# *RSC Advances* Outstanding Student Paper Awards 2022

**DOI:** 10.1039/d3ra90059b

**Published:** 2023-07-17

**Authors:** Laura Fisher

## Abstract

We are delighted to announce the winners for the *RSC Advances* Outstanding Student Paper Awards 2022. These awards recognise outstanding work published in the journal, for which a substantial component of the research was conducted by a student.
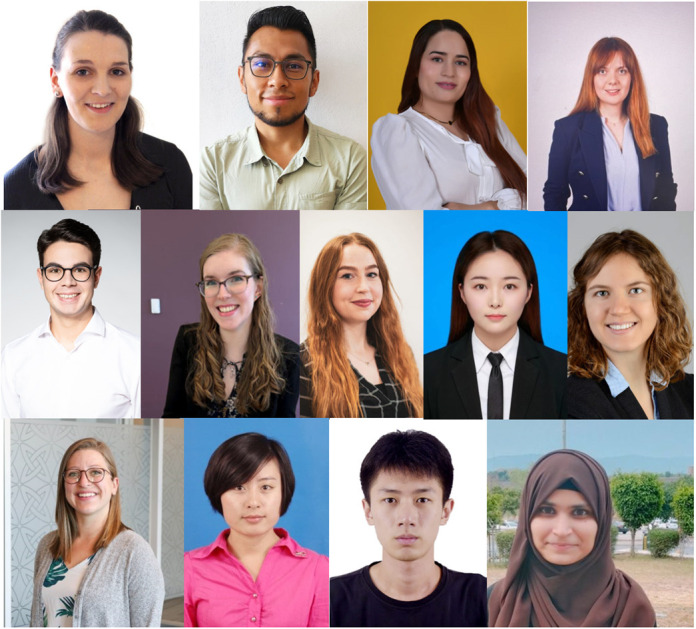

We are delighted to announce the winners for the *RSC Advances* Outstanding Student Paper Awards 2022. Following the success of our 2021 edition, *RSC Advances* will present an annual award series to recognise the hard work of students within the chemistry community.

All research articles published in *RSC Advances* in 2022 were considered. In order to be eligible for this award, the first author or co-first author must have been a student at the time of carrying out the research. From the support of corresponding authors, we received over 550 nominations highlighting the incredible talent and potential within the next generation of chemists. It was particularly inspiring to learn the exceptional works from diverse research fields and countries, a testament to the quality of work and curiosity throughout the community.

The nominations were shortlisted based on a number of criteria, and the winning papers were then selected by our Editorial Board and Associate Editors (Table 1).

**Table 1:** Winners of the Outstanding Student Paper Awards 2022

*Analytical Chemistry*	Margaret MacConnachie, Queen’s University, Canada
*Biological and Medicinal Chemistry*	Toni Pringle, Newcastle University, UK
*Catalysis*	Gen Li, Dalian University of Technology, China
*Computational & Theoretical Chemistry*	Stephanie Linker & Christian Schellhaas, ETH Zürich, Switzerland
*Energy Chemistry*	Karina Asheim, NTNU, Norway
*Environmental Chemistry*	Cui Li, China University of Geosciences, China
*Food Chemistry*	Xingyu Ding, Nanjing Tech University, China
*Inorganic Chemistry*	Nicole DiBlasi, University of Notre Dame, USA
*Materials Chemistry*	Despoina Eleftheriadou, UCL, UK
*Nanoscience*	Rabia Tahir, NUST, Pakistan
*Organic Chemistry*	Alejandro O. Viviano-Posadas, National Autonomous University of Mexico, Mexico
*Physical Chemistry*	Rawia Msalmi, Sfax University, Tunisia

Below, we highlight the winner of each subject category, and the research paper that won them the award. Please join us in congratulating all of our winners for their exceptional achievement. We look forward to witnessing their continued growth and impact as they embark on a promising career in the field of chemistry.

## Analytical Chemistry

**Margaret MacConnachie, Queen’s University, Canada** 
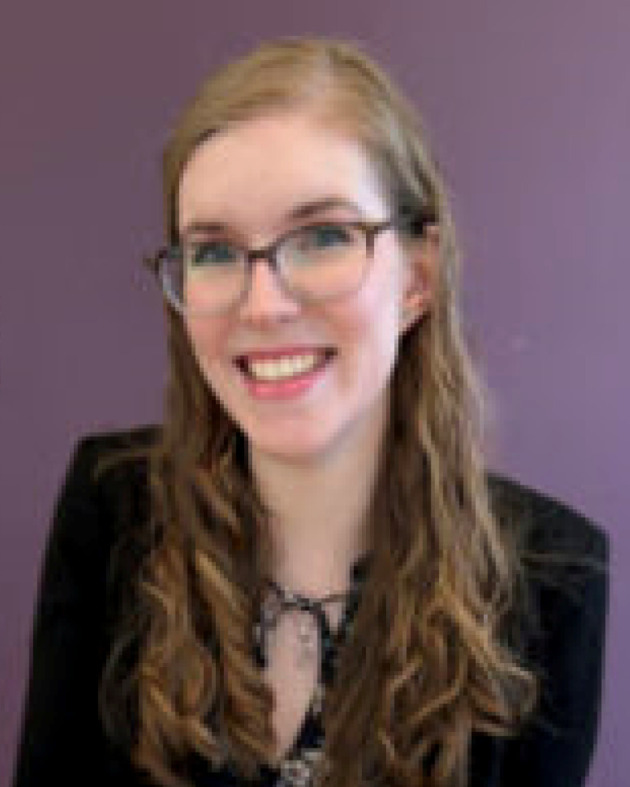


Margaret is recognised for her outstanding contribution in the research advance presented in:

*Sex determination of mummies through multi-elemental analysis of head hair using electrothermal vaporization coupled to inductively coupled plasma optical emission spectrometry* (https://doi.org/10.1039/D2RA05654B)

Originally hailing from Alberta, Canada, Margaret moved to Ontario to pursue her bachelor’s degree at Queen’s University. She received her BScH in 2018, with a major in chemistry and a minor in classical studies. During the last year of her degree, she completed a fourth-year research project on the analysis of solder for applications in forensic science, which sparked her interest in analytical chemistry. Following the completion of her undergraduate work, she stayed at Queen’s University to complete a PhD under the supervision of Dr Diane Beauchemin, working on projects which combine elemental analysis with both forensic and archaeological sciences. She recently submitted her thesis, titled ‘Novel Forensic and Archaeological Applications of Methods Involving the Direct Multi-Elemental Analysis of Solid Materials’. In the last year of her doctoral program, she received a MITACS Globalink Research Award which allowed her to spend six months working in an archaeometry research group at the University of Southern Denmark (Odense campus). Although interested in many areas of analytical chemistry, she has a particular passion for the intersection between chemical analysis, cultural heritage, and archaeology.

## Biological and Medicinal Chemistry

**Toni Pringle, Newcastle University, UK** 
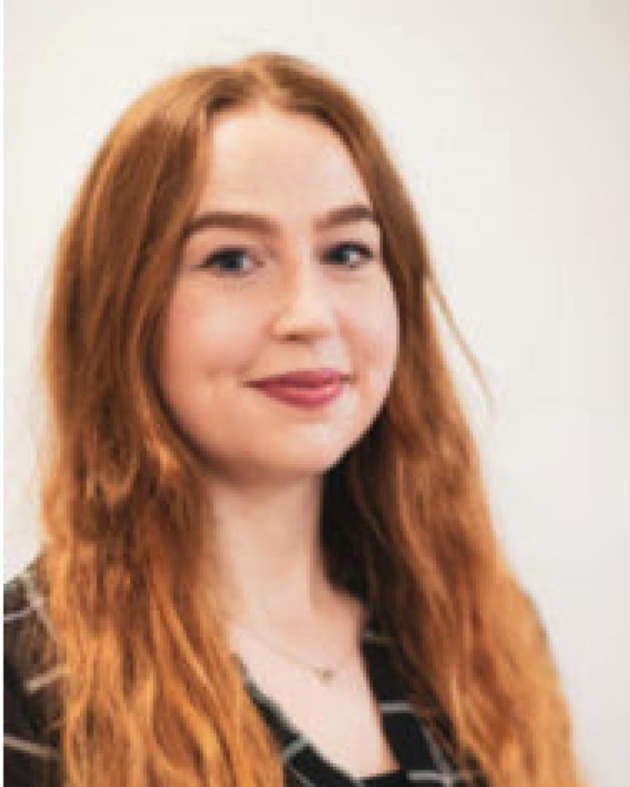


Toni is recognised for her outstanding contribution in the research advance presented in:

*The influence of degree of labelling upon cellular internalisation of antibody-cell penetrating peptide conjugates* (https://doi.org/10.1039/D2RA05274A)

Toni is a 4th year postgraduate researcher working with Dr James Knight at Newcastle University. Her research focuses on synthesis and preclinical evaluation of radioimmunoconjugates for positron emission tomography and fluorescence imaging, and cancer therapy. This includes the development of dual-modal antibody constructs for pre- and intra-operative imaging of sarcoma to enhance both surgical planning and the identification of tumour margins. She is also developing novel antibody constructs with cell-internalising properties for both diagnosis and therapy of cancer. Her current position follows the award of a 1st class MChem (Hons) degree in Chemistry with Medicinal Chemistry from Newcastle University. In her spare time, she enjoys hiking in the Lake District, snowboarding and sailing.

## Catalysis

**Gen Li, Dalian University of Technology, China** 
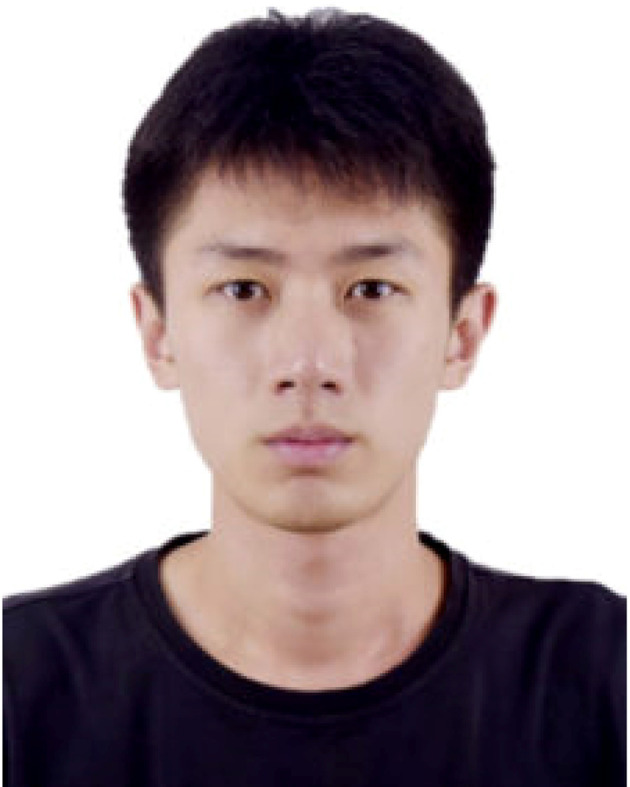


Gen is recognised for his outstanding contribution in the research advance presented in:

*Highly dispersed ruthenium nanoparticles on nitrogen doped carbon toward efficient hydrogen evolution in both alkaline and acidic electrolytes* (https://doi.org/10.1039/D2RA02671F)

Gen Li obtained his BS degree (2019) and MS (2022) degree in chemical engineering from Dalian University of Technology. He is now a PhD student at the Dalian University of Technology under the supervision of Professor Yujiang Song. His current research mainly focus on electrocatalysts and membrane electrode assembly toward hydrogen evolution reaction and/or oxygen evolution reaction in polymer electrolyte membrane water electrolysis.

## Computational & Theoretical Chemistry

**Stephanie Linker & Christian Schellhaas, ETH Zürich, Switzerland** 
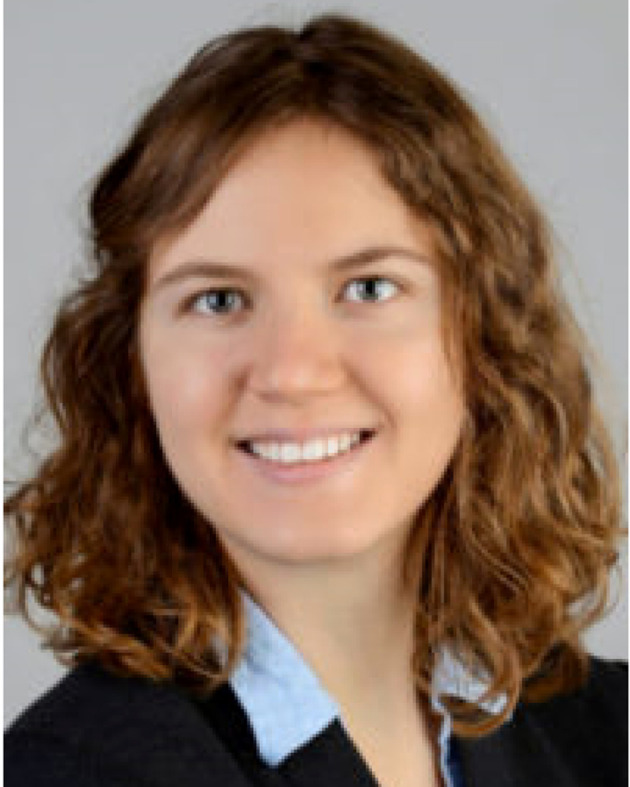
 
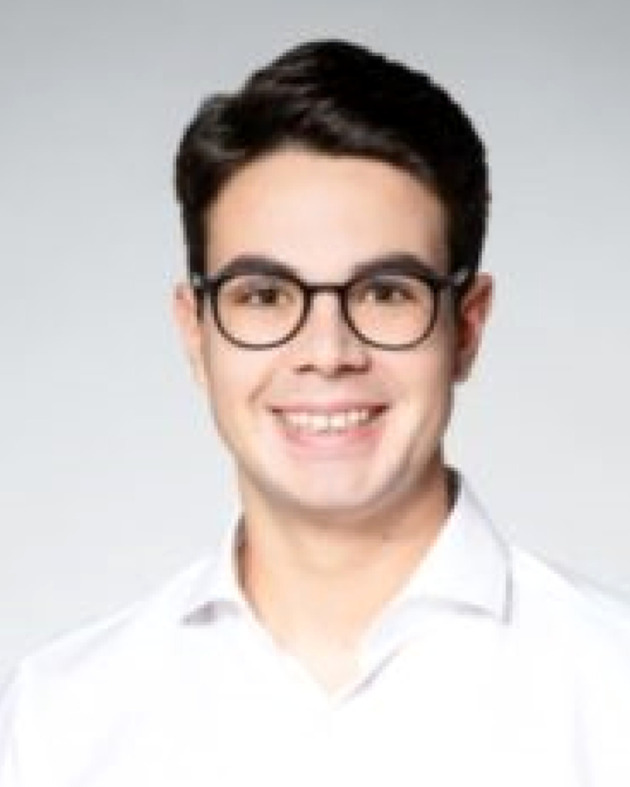


Stephanie and Christian are recognised for their outstanding contribution in the research advance presented in:

*Polar/apolar interfaces modulate the conformational behavior of cyclic peptides with impact on their passive membrane permeability* (https://doi.org/10.1039/D1RA09025A)

Stephanie holds a double degree in Biochemistry and Biophysics from the Goethe University in Frankfurt, Germany. In her studies she focused on the development of computational models for complex biological processes. Afterwards, Stephanie joined the Computational Chemistry group of Prof. Riniker at ETH Zurich, Switzerland for her PhD. There she used molecular dynamics simulations to study the permeability mechanism of large drug molecules. After defending her PhD in January 2023, Stephanie joined Merck (EMD) as a Computational Chemist. Beyond her academic achievements, Stephanie is a passionate advocate for science and is active on the board of the Swiss chemical society where she is responsible for international collaborations.

Christian studied Interdisciplinary Sciences with a focus on Chemical Biology and Theoretical Chemistry at ETH Zurich. He is currently pursuing his PhD in the field of protein engineering under the supervision of Prof. Bruno Correia at EPFL Lausanne. Initially, Christian started working on the computational design of proteins during his master’s thesis project in the research group of Prof. Possu Huang at Stanford University. Inspired by the work on the conformational behaviour of cyclic peptides, his current research interest focuses on the conformational dynamics of proteins and how these dynamics can inform the design of binding proteins. In his leisure time, Christian likes to play tennis and to make the most of the Swiss Alps, be it by hiking in summer or skiing in winter.

## Energy Chemistry

**Karina Asheim, Norwegian University of Science and Technology, Norway** 
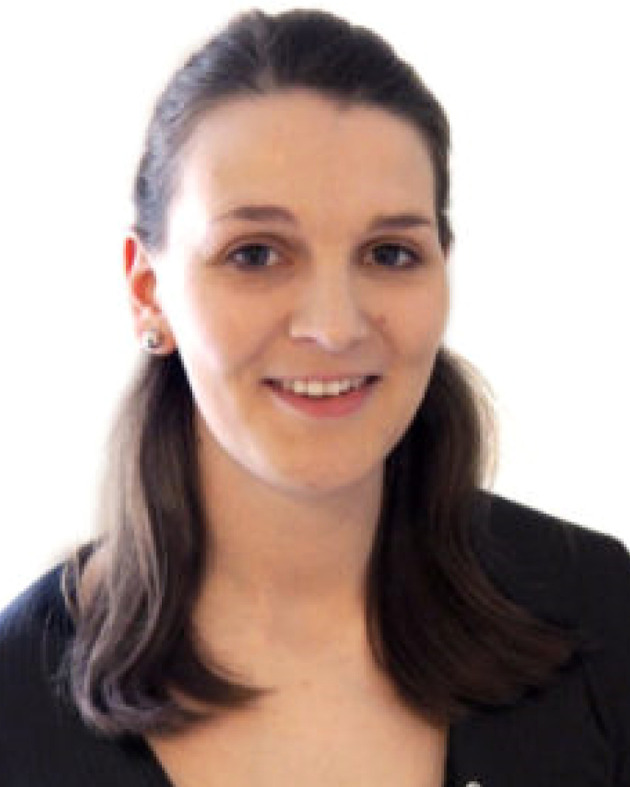


Karina is recognised for her outstanding contribution in the research advance presented in:

*Improved electrochemical performance and solid electrolyte interphase properties of electrolytes based on lithium bis(fluorosulfonyl)imide for high content silicon anodes* (https://doi.org/10.1039/D2RA01233B)

Karina graduated from a 5 year Master’s program in chemical engineering at the Norwegian University of Science and Technology in 2016. During the study she specialized in materials science and materials for energy technology, finishing with a Master Thesis on Mg-ion batteries. Continuing in the world of batteries, she started on a PhD project in Li-ion batteries where the work focussed on electrolyte for silicon-based anodes. The work was supervised by Prof. Ann Mari Svensson, was carried out at the Norwegian University of Science and Technology, and was completed in 2021. Now Karina works on battery separators for a Norwegian polymer R&D company called Norner AS.

## Environmental Chemistry

**Cui Li, China University of Geosciences, China** 
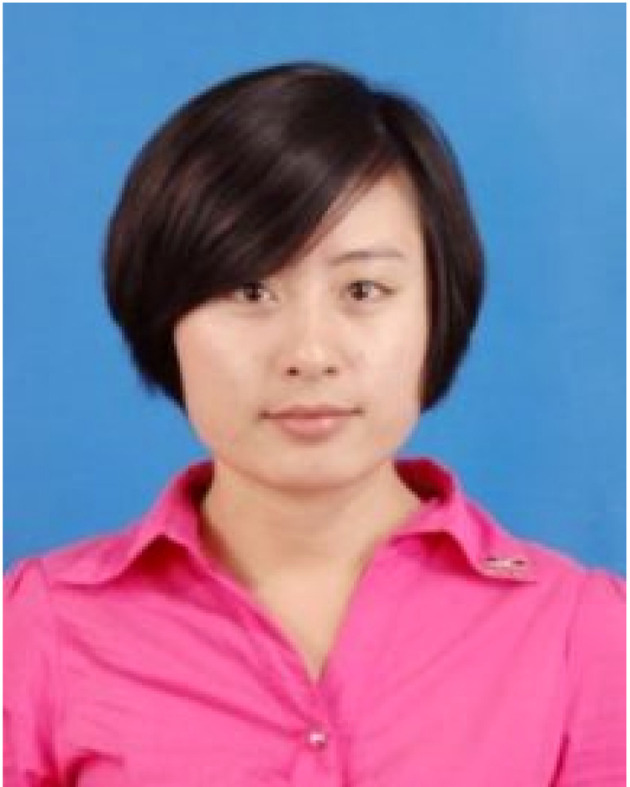


Cui is recognised for her outstanding contribution in the research advance presented in:

*Response of chlorinated hydrocarbon transformation and microbial community structure in an aquifer to joint H*_*2*_ *and O*_*2*_ (https://doi.org/10.1039/D2RA04185E)

Cui Li, doctor from China University of Geosciences, majoring in Environmental Science and Engineering. The research direction focuses on microorganisms, with the goal of achieving efficient treatment of pollutants. The main research is environmental microbiology, microbial community function and its transformation mechanism of organic pollutants. Published 3 SCI papers and applied for 2 utility model patents during the doctoral period.

## Food Chemistry

**Xingyu Ding, Nanjing Tech University, China** 
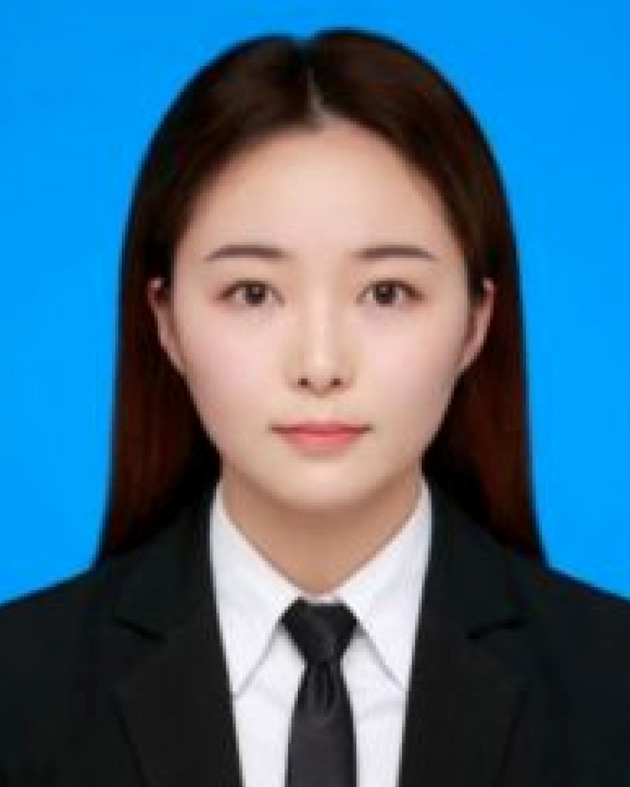


Xingyu is recognised for her outstanding contribution in the research advance presented in:

*Preparation of chitosan-coated polystyrene microspheres for the analysis of trace Pb(* *ii* *) ions in salt by GF-AAS assisted with solid-phase extraction* (https://doi.org/10.1039/D2RA04968F)

Ding Xingyu was born in Jiangsu Province, China in 1997. Her bachelor’s and master’s degrees were obtained from Nanjing University of Technology under the supervision of Associate Professor Li Yi, and all of her research achievements are inseparable from his guidance and assistance. Her research direction is food safety, and the title of her master’s project is “Research and Preparation of Novel Polymer Carriers for Concentration and Enrichment of Harmful Heavy Metal Ions”.

This article, “Preparation of chitosan-coated polystyrene microspheres for the analysis of trace Pb(ii) ions in salt by GF-AAS assisted with solid-phase extraction”, focuses on the enrichment and analysis of harmful heavy metal lead in food. The article studies and prepares polymer microspheres to overcome the interference of a high salt background and achieve the separation and detection of heavy metal lead.

In the future, food safety will receive increasing attention, and the enrichment materials and detection methods for heavy metals will also show diversified development. Xingyu hopes that the food safety industry will flourish and more scholars will join in.

## Inorganic Chemistry

**Nicole DiBlasi, University of Notre Dame, USA** 
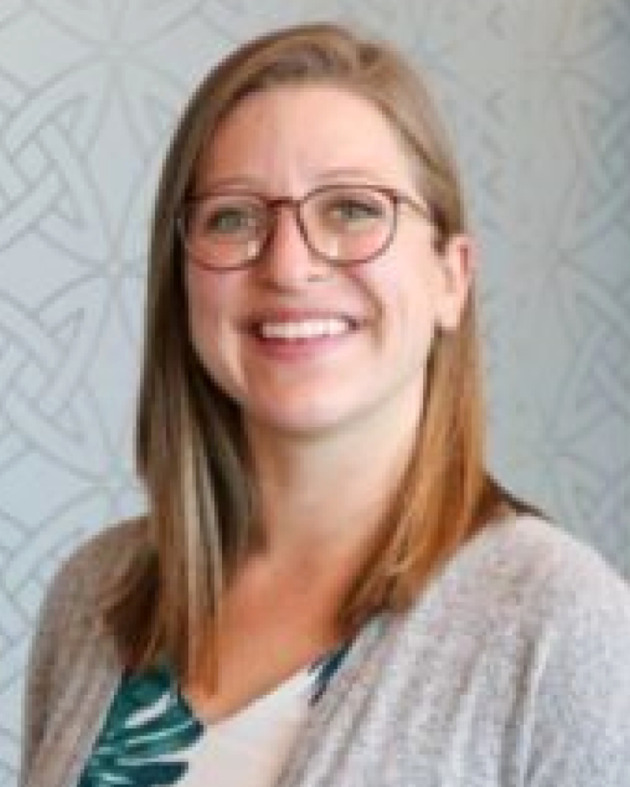


Nicole is recognised for her outstanding contribution in the research advance presented in:

*Pu(* *iii* *) and Cm(* *iii* *) in the presence of EDTA: aqueous speciation, redox behavior, and the impact of Ca(* *ii* *)* (https://doi.org/10.1039/D1RA09010K)

Nicole A. DiBlasi is a Scientist in Actinide Analytical Chemistry at Los Alamos National Lab with 8 years of experience in actinide and environmental radiochemistry. After receiving her bachelor’s in chemistry from the University of Missouri in 2016, Nicole pursued her doctorate in actinide chemistry at the University of Notre Dame under the guidance of Dr Amy E. Hixon where her doctoral research focused on the speciation, solubility, and redox behaviour of the Pu–EDTA system under conditions relevant for deep geological repositories. In addition to her dissertation work, Nicole was able to participate in other projects including work with novel actinide compound synthesis and characterization and the development of synthesis methods for post-detonation nuclear melt glass reference materials for use in nuclear forensics. Following the completion of her PhD in 2021, Nicole became a postdoctoral research associate at the Institute for Nuclear Waste Disposal at the Karlsruhe Institute of Technology in Karlsruhe, Germany, where she performed research on actinide– and technetium–organic interactions under alkaline and high ionic strength conditions. In late 2022, Nicole accepted a position as a scientist at Los Alamos National Laboratory where she performs high accuracy, high precision analyses on actinide materials as part of the Radiochemistry team in the Actinide Analytical Chemistry group.

## Materials Chemistry

**Despoina Eleftheriadou, University College London, UK** 
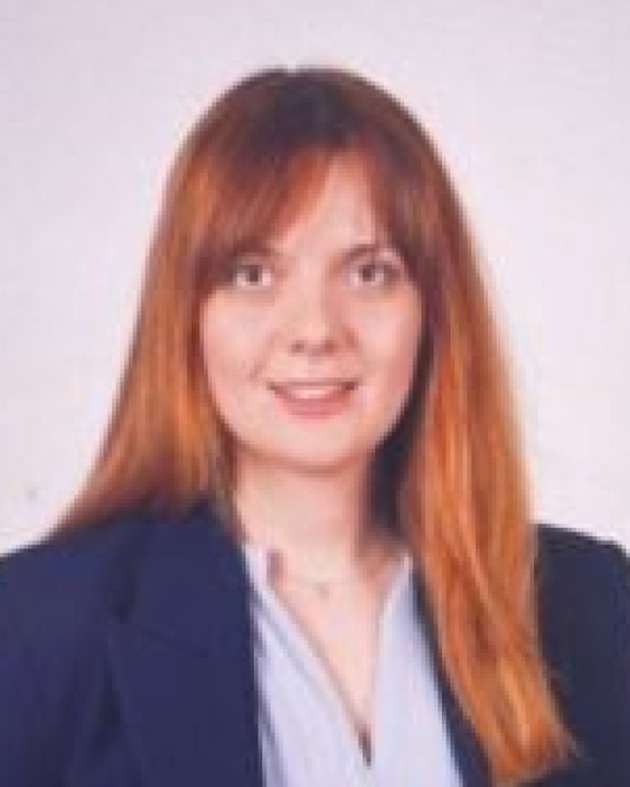


Despoina is recognised for her outstanding contribution in the research advance presented in:

*An alginate-based encapsulation system for delivery of therapeutic cells to the CNS* (https://doi.org/10.1039/D1RA08563H)

Despoina Eleftheriadou obtained her MEng in Chemical Engineering from the Aristotle University of Thessaloniki in 2016. She then completed her MSc in Nanotechnology and Regenerative Medicine at University College London in 2018. During this time, she was able to work on various projects including nanobiomaterials for Alzheimer’s disease treatment and immunomodulation for therapeutic cell transplantation in the CNS. She is currently a PhD student at the University College London Centre for Nerve Engineering, focusing on mathematical modelling led design of nerve repair constructs. Her research interest lies in working at the interface of engineering and life sciences.

## Nanoscience

**Rabia Tahir, National University of Sciences and Technology, Pakistan** 
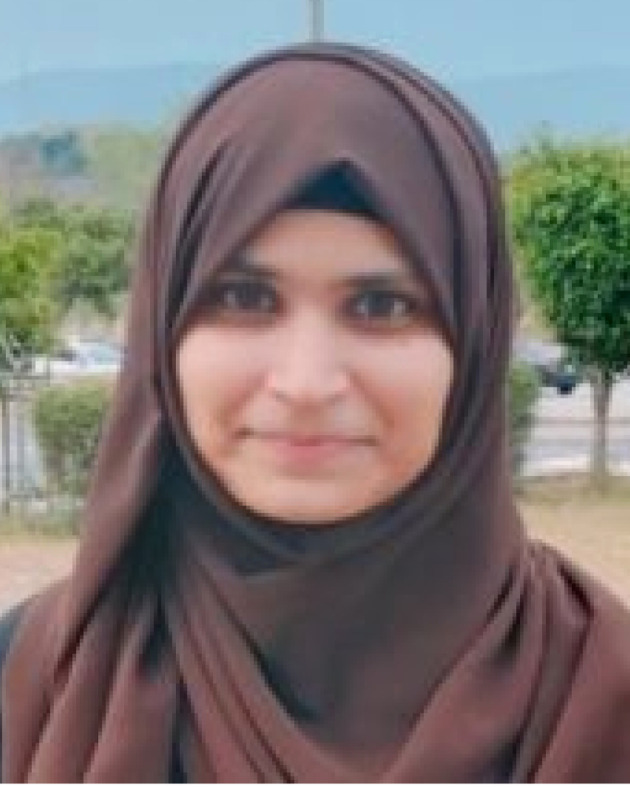


Rabia is recognised for her outstanding contribution in the research advance presented in:

*First observation on emergence of strong room-temperature ferroelectricity and multiferroicity in 2D-Ti*_*3*_ *C*_*2*_ *T*_*x*_ *free-standing MXene film* (https://doi.org/10.1039/D2RA04428E)

Born in Multan (southern Punjab city in Pakistan) and completed her Bachelor’s degree from The Women University Multan (WUM), Rabia Tahir is currently enrolled as a PhD student at the Department of Physics, School of Natural Sciences (SNS), National University of Sciences and Technology (NUST), Islamabad, Pakistan. Under the expert guidance of her supervisor, Prof. Dr Syed Rizwan, she has embarked on a journey to explore the unique properties and potential applications of 2D materials such as MXene and their potential as a ferroelectric and multiferroic material that has been a long-standing issue of interest in the research community. Rabia Tahir reported the ferroelectricity and multiferroicity in 2D Ti_3_C_2_T_*x*_ MXene for the first time that may revolutionize next-generation data storage devices with enhanced functionalities.

Rabia Tahir is also hired as a research associate under the Higher Education Commission (HEC) of Pakistan under project No. 20-14784/NRPU/R&D/HEC/2021. In her leisure time, she likes to play sports and reads books with inspiring lessons.

## Organic Chemistry

**Alejandro O. Viviano-Posadas, National Autonomous University of Mexico, Mexico** 
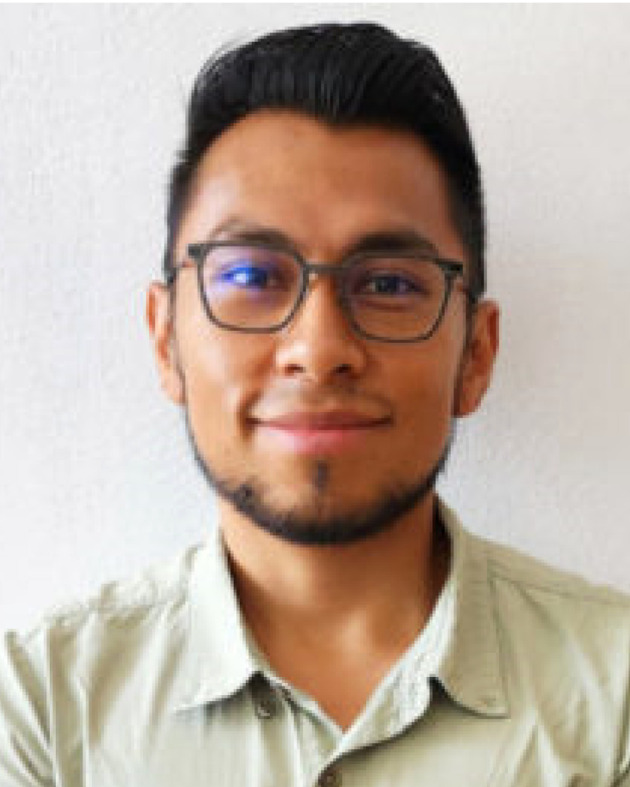


Alejandro is recognised for his outstanding contribution in the research advance presented in:

*Efficient fluorescent recognition of ATP/GTP by a water-soluble bisquinolinium pyridine-2,6-dicarboxamide compound. Crystal structures, spectroscopic studies and interaction mode with DNA* (https://doi.org/10.1039/D2RA05040D)

Alejandro Viviano was born in Mexico City. He received his BSc (2019) and MSc (2021) degrees with theses focused on the synthesis and luminescent properties of novel Pd/Pt-based complexes with analytical applications. He is currently pursuing his PhD with Professor Alejandro Dorazco at the Chemistry Institute from the National Autonomous University of Mexico.

His research involves the molecular recognition of neurotransmitters and nucleotides using novel organometallic and organic receptors. To date, their scientific results have been published in seven research articles (ORCID: 0000-0002-3588-5836).

## Physical Chemistry

**Rawia Msalmi, Sfax University, Tunisia** 
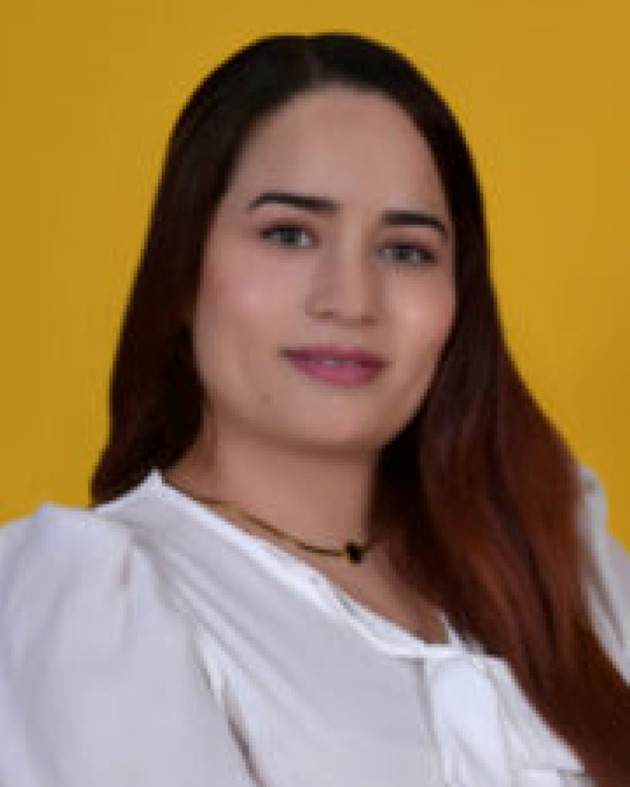


Rawia is recognised for her outstanding contribution in the research advance presented in:

*Organically tuned white-light emission from two zero-dimensional Cd-based hybrids* (https://doi.org/10.1039/D1RA08953F)

Dr Rawia Msalmi obtained a PhD degree in Chemistry from the Faculty of Sciences of Sfax, Sfax University, Tunisia. For her thesis, she performed the physico-chemical characterization of Cd- and Pb-based white light emitter hybrid materials (WLEHMs). Her research focus was on the contribution of the organic molecules and the tridimensional assembly mode in the performance of the emitted white light. She has published the findings of her PhD study in four peer-reviewed journal articles. In line with her PhD work, she contributed in other research papers on the study of the optical behavior of one-dimensional Cu-based perovskites published in *Journal of Materials Chemistry C* as second co-author.

Dr Rawia Msalmi is currently a postdoctoral researcher in the Laboratory of Physico-Chemistry of the Solid State, Chemistry Department, Faculty of Sciences of Sfax, Sfax University, Tunisia, under the supervision of Professor Houcine Naïli. Her present investigation focuses on the stabilization and physico-chemical characterization of lead-free hybrid materials for environmentally friendly photovoltaic solar cells and lighting sources. She co-supervises research activities in the same field. Thus far, she has 10 publications in high impact journals.

Please join us in congratulating all of our winners for their exceptional achievement. We extend our sincere gratitude to all the authors for their contributions, as well as to the editors and reviewers for their collaboration, which has resulted in this high-quality series.

We will continue to recognise outstanding student contributions and plan to give out these awards each year. If you published a research article in 2023, or go on to publish with the journal in the future, and would like to recognise a significant contribution made by a student, we invite them to join us in future editions of this series. Please email advances-rsc@rsc.org for more information.

Dr Laura Fisher, Executive Editor for *RSC Advances*

## Supplementary Material

